# Effect of 10 Week Beta-Alanine Supplementation on Competition and Training Performance in Elite Swimmers

**DOI:** 10.3390/nu4101441

**Published:** 2012-10-09

**Authors:** Weiliang Chung, Greg Shaw, Megan E. Anderson, David B. Pyne, Philo U. Saunders, David J. Bishop, Louise M. Burke

**Affiliations:** 1 Department of Physiology, Australian Institute of Sport, Leverrier Crescent, Bruce, Canberra, ACT 2617, Australia; Email: weiliang.chung@hotmail.com (W.C.); philo.saunders@ausport.gov.au (P.U.S.); 2 Institute of Sport, Exercise and Active Living (ISEAL), Victoria University, Ballarat Road, Footscray Park, Melbourne, VIC 3011, Australia; Email: david.bishop@vu.edu.au; 3 Department of Sports Nutrition, Australian Institute of Sport, Leverrier Crescent, Bruce, Canberra, ACT 2617, Australia; Email: greg.shaw@ausport.gov.au (G.S.); louise.burke@ausport.gov.au (L.M.B.); 4 Department of Physiology, Queensland Academy of Sport, Level 1 Queensland Sport and Athletics Centre (QSAC), Kessels Road, Nathan, Brisbane, QLD 4111, Australia; Email: megan.anderson@communities.qld.gov.au

**Keywords:** carnosine, physiology, ergogenic aid, swimming, exercise, athlete

## Abstract

Although some laboratory-based studies show an ergogenic effect with beta-alanine supplementation, there is a lack of field-based research in training and competition settings. Elite/Sub-elite swimmers (*n* = 23 males and 18 females, age = 21.7 ± 2.8 years; mean ± SD) were supplemented with either beta-alanine (4 weeks loading phase of 4.8 g/day and 3.2 g/day thereafter) or placebo for 10 weeks. Competition performance times were log-transformed, then evaluated before (National Championships) and after (international or national selection meet) supplementation. Swimmers also completed three standardized training sets at baseline, 4 and 10 weeks of supplementation. Capillary blood was analyzed for pH, bicarbonate and lactate concentration in both competition and training. There was an unclear effect (0.4%; ±0.8%, mean, ±90% confidence limits) of beta-alanine on competition performance compared to placebo with no meaningful changes in blood chemistry. While there was a transient improvement on training performance after 4 weeks with beta-alanine (−1.3%; ±1.0%), there was an unclear effect at ten weeks (−0.2%; ±1.5%) and no meaningful changes in blood chemistry. Beta-alanine supplementation appears to have minimal effect on swimming performance in non-laboratory controlled real-world training and competition settings.

## 1. Introduction

The use of dietary supplements is common in athletic populations [1]. Examples of supplements which enjoy popular support, as well as a strong evidence-base for ergogenic effects on sports performance, include creatine, caffeine and sodium bicarbonate [2,3]. A newer supplement of interest among both athletes and sports scientists, is the amino acid, beta-alanine. This interest stems from recent evidence that chronic beta-alanine supplementation can increase carnosine concentration in the muscle [4,5,6]. 

Beta-alanine is a precursor of carnosine (beta-alanyl-L-histidine), an important muscle compound which, among other roles, is estimated to account for ~6%–7% of intracellular buffering capacity in the human vastus lateralis muscle [7,8]. It should be noted that this value may underestimate the true role of carnosine in muscle buffering capacity, due to an artifact in the measurement technique in which the homogenization of muscle samples exposes pH active compounds that would otherwise be unavailable within the cell [9].

The effectiveness of carnosine as an intracellular buffer is related to its imidazole ring having a pKA of 6.83, close to intracellular pH [10]. Beta-alanine has been identified as the limiting factor in carnosine synthesis [11] and supplementing dietary intake with 4 to 6.4 g/day of beta-alanine has been shown to increase muscle carnosine concentrations by 59% after 4 weeks and 80% after 10 weeks [5]. 

An increase in muscle carnosine concentration following beta-alanine supplementation may improve performance during exercise tasks associated with the accumulation of hydrogen ions (H^+^). In support of this assertion, studies have reported ergogenic benefits of beta-alanine supplementation in a variety of laboratory-based protocols, including a 30-s maximal effort at the end of a simulated cycling race [12], repeated maximal isokinetic knee extensions [6], and total work done in a cycle to exhaustion test [5]. More recently, Baguet and colleagues [13] investigated the effects of beta-alanine supplementation on 2000-m rowing ergometer performance. After 7 weeks of supplementation, the beta-alanine group improved their performance by 2.7 s, to be 4.3 s quicker than the placebo group. Although this difference did not reach statistical significance (*p* = 0.07), it is very likely to be worthwhile in elite sporting competitions, where the margins between winning the gold or silver are small (e.g., 0.8 s in the men’s single scull rowing event at the 2008 Beijing Olympic Games). 

However, a limitation of the existing research on beta-alanine supplementation is that most studies have involved non-elite participants and laboratory-based performance tests which do not necessarily relate to elite level sport. The results from these highly-controlled laboratory studies may not necessarily reflect the variability of performances in the field attributable to factors such as training programs, diet, residual fatigue, underlying soreness or injury, and/or the level of motivation of the athlete. Therefore, any transfer of these positive laboratory-based effects needs to be investigated within an applied setting. To our knowledge, this is the first study to examine the effectiveness of beta-alanine supplementation in elite athletes on training and competition in a real-world setting. 

## 2. Experimental Section

**Figure 1:**
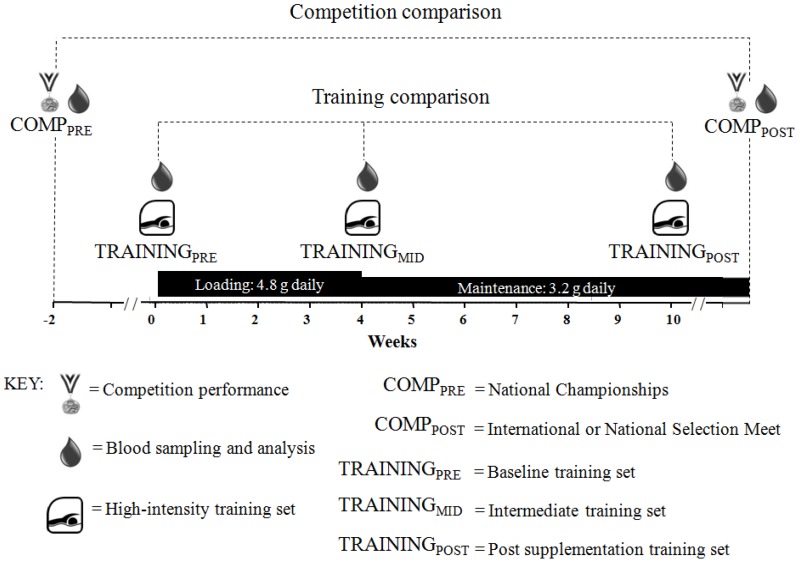
Schematic timeline of study design.

A placebo-controlled, double-blind study was performed to investigate the effect of beta-alanine supplementation in swimmers on competition and training performance (Figure 1). The effect of 10 weeks of beta-alanine supplementation on competition performance was determined by changes in post-tapered race performance between the 2011 Australian Swimming Championships (COMP_PRE_) and the 2011 World Championships, World University Games, or national selection meeting (COMP_POST_).

We also determined the effect of beta-alanine supplementation on performance during swim training. Swimmers were asked to complete one of three pool training sets, specific to their race distance on three occasions; at baseline (TRAINING_PRE_), four weeks (TRAINING_MID_) and ten weeks of supplementation (TRAINING_POST_). These training sets were designed by the researchers in consultation with experienced, international-level swim coaches and are typical of those undertaken regularly by the elite swimmers in this study. 

This multi-center trial was conducted by a team of sport scientists based in four different states and territories (Australian Capital Territory, New South Wales, Queensland and Victoria) and involved 14 different swim squads. Due to practicality and the desire to investigate whether any effects of beta-alanine were robust in relation to real-world conditions, we did not attempt to control training programs or diet over the course of this study. However, control was required on use of other dietary supplements (e.g., creatine, caffeine and sodium bicarbonate) during the period of study. The study was approved by the Ethics Committee of the Australian Institute of Sport (approval number 20110209) and all swimmers in the study provided written informed consent.

### 2.1. Subjects

**Figure 2:**
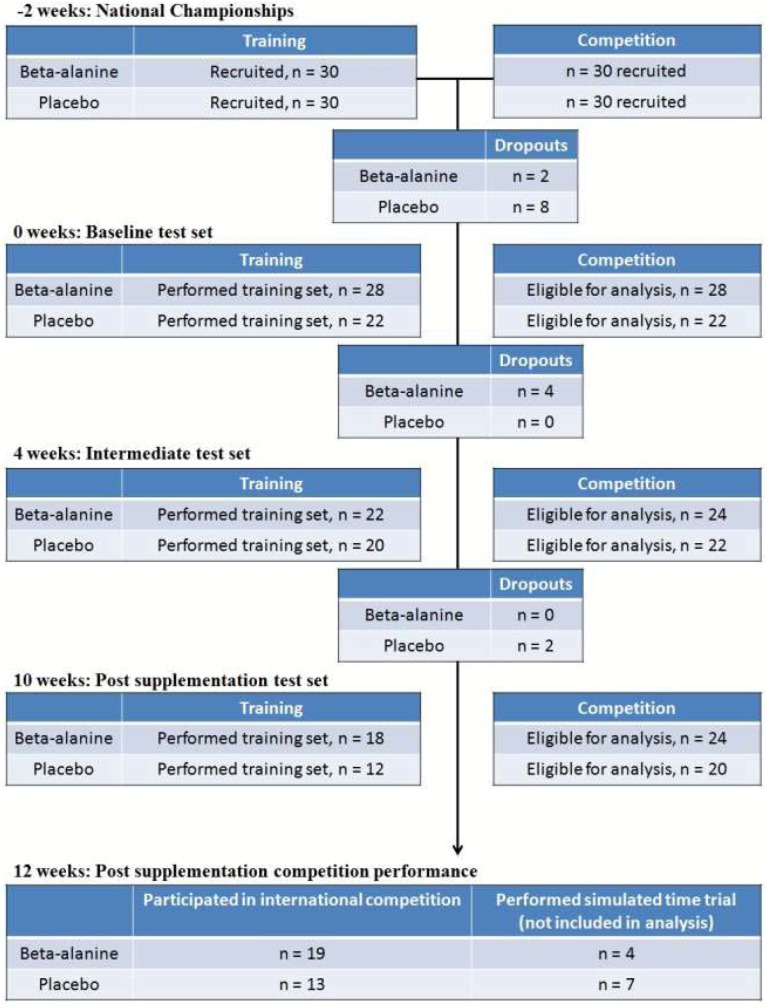
Changes in subject numbers and International Point Score (IPS) profiles over the course of the study. IPS reported as mean ± SD refers to the mean International Point Score as a standardized measure of competition performance.

A total of 34 male and 26 female elite/sub-elite swimmers, representing all competitive swim strokes and race distances, volunteered to participate in this study. We estimated that a total sample size of 42 swimmers (7 swimmers in each intervention group) would be sufficient to detect a change of 1% improvement in performance. Of the initial 60 swimmers with baseline competition data, 10 swimmers dropped out of the study prior to supplementation due to injury and other reasons not associated with the study. The dropout of swimmers for the study is reported in Figure 2. 

Swimmers were assigned into one of three training groups (Sprint: 50-m to 100-m specialists, Middle distance: 100-m to 200-m specialists, and Distance: 200-m to 400-m specialists) and ranked according to the International Point Score (IPS) of their best competition performance. The IPS system is used by the international governing body of swimming, Fédération International de Natation (FINA), to standardize competition performances between different sex, swim strokes and race distances. Following the ranking, each swimmer was pair-matched for sex, race distance and performance level, and then randomly assigned to either the beta-alanine (BA) or placebo (PLA) supplementation group. The swimmers performed both the training and competition aspects of this study using their best competitive swim stroke. Information on weekly training volumes for each swimmer was obtained from coaches log books. The weekly training volume over the study period was 40 ± 4 km (mean ± SD) with a reduction of ~33% in volume from the peak training week (9 weeks prior to the final competition) to the final week of the taper.

### 2.2. Supplementation

Swimmers were asked to commence supplementation after baseline data collection. The supplementation protocol involved ingesting 4.8 g per day (two 800 mg tablets, three times daily) of beta-alanine or placebo with meals for 4 weeks (loading phase). At this point, the swimmers were asked to return all unconsumed tablets to monitor compliance, before being provided with 3.2 g/day (two tablets, twice daily) of beta-alanine or placebo for the remainder of the study period (maintenance phase). All supplements were packaged in identical containers, labeled and couriered to each center by a researcher who had no direct involvement with any participating swim squad or coach.

At the end of the study, all remaining supplements were required to be returned to account for compliance. Swimmers were also asked to participate in an online survey to provide feedback on supplementation blinding and side effects. The total dosage of beta-alanine consumed in this study is similar to protocols that have been previously shown to increase muscle carnosine stores [5,14]. 

Since inadvertent doping is always a concern with dietary supplementation and elite athletes, beta-alanine was sourced from a company maintaining good manufacturing practices and involved in a contamination control program (Sustained Release Beta-alanine, Musashi, Australia). Because this company was unable to provide a suitable placebo for their product, we organized for the beta-alanine tablets to be re-pressed by an accredited pharmaceutical vendor (GMP Pharmaceuticals, NSW, Australia) to achieve an identical appearance to a placebo tablet made of maltodextrin. 

### 2.3. Competition Analysis

The mean difference in log-transformed performance times between COMP_PRE_ and COMP_POST_ was used to compare competition performance between BA and PLA. Capillary blood (200 μL) was sampled from a fingertip or earlobe prior to warm up and 6 min after each race. The blood samples were analyzed immediately using a portable blood-gas analyzer (i-STAT, Abott, IL, USA) to determine pH and bicarbonate concentrations. The i-STAT analyzer provides reliable measures of blood pH and bicarbonate across a range of exercise intensities [15]. The typical errors (absolute units and relative %) in our laboratory are: pH (0.01, 0.1%); bicarbonate (0.38 mmol/L, 1.8%) and lactate concentration (0.10 mmol/L, 2.7%). We were unable to report lactate concentration during competition as the i-STAT analyzer does not provide a sensitive measure of values above 20 mmol/L, which commonly occur in this situation.

### 2.4. Training Analysis

Differences at baseline (TRAINING_PRE_), four weeks (TRAINING_MID_) and ten weeks of supplementation (TRAINING_POST_) were used to compare swim training performance. Swim training performance was measured as the sum of the mean times of the combined 50-m swims and the all out effort at the end of each training set. The distance-specific training set (described below) consisted of repeated maximal 50-m efforts immediately followed by a distance specific maximal effort. The combination of the repeat 50’s and distance specific maximal effort was to ensure a significant level of cellular fatigue was achieved allowing elucidation of the full potential of beta-alanine supplementation on high-intensity training in a fatigued state.

Sprint: 4 × 50-m on a 3 min cycle (maximal) + 100-m maximal effort 

Middle distance: 6 × 50-m on a 2 min cycle (maximal) + 200-m maximal effort

Distance: 8 × 50-m on a 1.5 min cycle (maximal) + 200-m maximal effort

During each training set, capillary blood was sampled from a fingertip prior to warm up and 6 min after the time trial. The blood samples were analyzed immediately using the i-STAT analyzer to determine pH and bicarbonate concentrations. In addition, another 5 µL blood sample was collected and analyzed immediately for lactate concentration at the same time points using a portable blood lactate analyzer (Lactate Pro, Arkray, Kyoto, Japan). We were unable to analyze all samples at all time points due to equipment failure or logistical reasons which resulted in different number of samples for the analyses of results.

### 2.5. Statistical Analysis

We employed a contemporary analytical approach involving magnitude-based inferences to detect small effects of practical importance in an elite athlete group. Specifically, magnitude-based inferences and precision of estimation expressed as 90% confidence limits (CL) were used to evaluate differences within and between groups [16]. All data are expressed as raw units in mean ± SD unless otherwise specified. Analysis of effects between groups were performed via log-transformed data to account for non-uniformity of error [17]. A reference value of 0.3% for the smallest worthwhile change was used to compare competition performance. Briefly, this value was derived from a third [17] of the typical within-swimmer race-to-race SD of 0.8% [18]. For measures not directly related to competition performance, the smallest worthwhile change was calculated as Cohen’s smallest standardized effect size (0.2 × between-athlete SD). The magnitudes of standardized differences between groups were interpreted as: trivial, <0.2; small, 0.2–0.6; moderate, 0.6–1.2 and large, >1.2. An outcome was deemed unclear when the confidence interval crossed limits for both a substantially positive and negative effect [17].

## 3. Results

### 3.1. Subject Characteristics and Supplementation

Of the 60 swimmers who volunteered for this study, 10 swimmers dropped out prior to supplementation due to injury or loss of interest, with 4 more swimmers dropping out after the first training set due to loss of interest (Figure 2). As a result of the culmination of dropouts, we were unable to utilize distance group as a covariant in the final analyses of this study. Therefore, we combined all three distance groups and assessed the data which included a total of 41 swimmers with two subsets of complete competition or training data (Table 1). 

Results of an online survey undertaken with 41 respondents upon conclusion of the study provided information on the success of blinding of treatments. In this regard, 12 out of 22 respondents who were in the BA group correctly guessed the identity of the supplement they received, with 10 of these swimmers reporting mild paraesthesia. For PLA, 12 out of 19 respondents guessed correctly, attributing it to the absence of side effects (5 respondents) and taste (3 respondents). The total dosage of beta-alanine and placebo ingested by the swimmers was 379 ± 51 g (mean ± SD) and 360 ± 54 g respectively.

**Table 1:** Characteristics of swimmers included in the final data analysis.

	Male	Female	Total	Age	Mass	IPS
	(*n*)	(*n*)	(*n*)	(year)	(kg)	(points)
**Competition (*n* = 32)**						
**Beta-alanine**	9	10	19	22.6 ± 2.8	74.3 ± 9.7	854 ± 52 *
**Placebo**	6	7	13	21.0 ± 2.4	67.4 ± 8.7	834 ± 72
**Training (*n* = 30)**						
**Beta-alanine**	11	7	18	21.8 ± 3.3	76.0 ± 8.5	846 ± 56 *
**Placebo**	6	6	12	21.3 ± 2.4	71.6 ± 11.3	816 ± 70

Data are mean ± SD. IPS = FINA international point score classification of swimming performance recorded at the National Championships in April 2011. * indicates small difference in baseline IPS between BA and PLA.

### 3.2. Competition Performance

Log-transformed race performance times for BA at baseline and post-supplementation (for sprint, middle-distance and distance groups combined) were 454.6 ± 52.5 (mean ± SD) and 454.6 ± 52.6, with PLA race performance times being 479.0 ± 70.4 and 478.6 ± 70.7, respectively. The differences in race performances between groups equated to an unclear effect of BA (0.4% ± 0.8%, mean; ±90% confidence limits).

### 3.3. Competition Blood pH and Bicarbonate

Due to equipment error, blood pH and bicarbonate samples from 5 swimmers have been excluded. There were no clear effects of beta-alanine supplementation on pre and post race blood pH. Similarly, there were no substantial effects of beta-alanine supplementation on pre and post race blood bicarbonate in all groups (Table 2).

**Table 2:** Unclear effects of beta-alanine (BA) supplementation on pre and post race blood pH and bicarbonate values between the pre- and post-supplementation competitions. A total of 27 blood samples were analyzed on both pre and post tests.

	Beta-alanine ( *n* = 16)		Placebo ( *n* = 11)	
	Pre-supplementation	Post-supplementation	Pre-supplementation	Post-supplementation
**pH**				
**Pre race**	7.48 ± 0.04	7.46 ± 0.05	7.48 ± 0.05	7.46 ± 0.05
**Post race**	7.11 ± 0.10	7.13 ± 0.11	7.13 ± 0.10	7.14 ± 0.13
**Bicarbonate (mmol** **/** **L)**				
**Pre race**	26.4 ± 3.0	26.5 ± 3.6	25.8 ± 4.0	25.7 ± 3.7
**Post race**	10.4 ± 3.7	10.7 ± 3.8	9.8 ± 2.6	11.1 ± 4.3

Data are mean ± SD.

### 3.4. Training Sets

**Figure 3:**
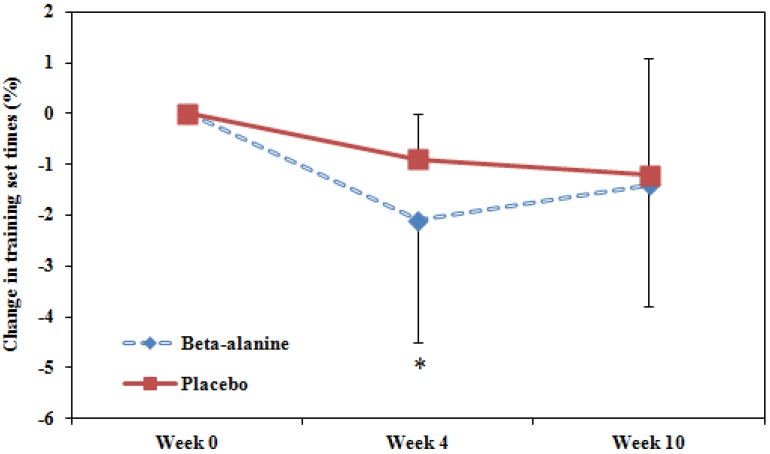
Beta-alanine supplementation improved training set times at 4 weeks but not 10 weeks. Data are mean ± SD. * indicates a substantial difference between BA and PLA.

There was a transient substantially greater improvement in training performance after 4 weeks in the beta-alanine group compared with the placebo group (−1.3%; ±1.0%, mean; ±90% CL) but unclear after 10 weeks (−0.2%; ±1.5%, see Figure 3).

### 3.5. Training Blood pH, Bicarbonate and Lactate

No substantial effects of beta-alanine supplementation were present in relation to pre and post training blood pH and bicarbonate after 4 and 10 weeks (Table 3). However, there was a small effect of beta-alanine supplementation on post-training blood lactate at 4 weeks (−12.2%; ±11.6%, mean ±90% CL) and 10 weeks (−10.0%; ±10.2%). 

**Table 3:** No substantial effect of BA on pre and post training values of blood pH, bicarbonate and lactate at baseline, 4 and 10 weeks post supplementation. A total of 30 blood samples were analyzed for pre and post training sets.

	Beta-alanine ( *n* = 18)			Placebo ( *n* = 12)		
	0 week	4 weeks	10 weeks	0 week	4 weeks	10 weeks
**pH**						
**Pre training**	7.46 ± 0.03	7.45 ± 0.03	7.44 ± 0.03	7.46 ± 0.03	7.44 ± 0.02	7.45 ± 0.03
**Post training**	7.20 ± 0.13	7.19 ± 0.13	7.18 ± 0.13	7.21 ± 0.08	7.18 ± 0.09	7.18 ± 0.07
**Bicarbonate (mmol** **/** **L)**						
**Pre training**	25.2 ± 1.9	25.5 ± 1.8	24.9 ± 1.5	24.2 ± 2.3	24.7 ± 1.8	24.2 ± 1.6
**Post training**	11.0 ± 3.2	10.8 ± 3.5	10.8 ± 3.2	10.8 ± 3.2	9.4 ± 2.7	10.3 ± 2.7
**Lactate (mmol** **/** **L)**						
**Post training**	11.9 ± 2.3	10.8 ± 2.2 ^$^	11.1 ± 2.1 *	11.7 ± 2.3	12.0 ± 1.6	12.1 ± 2.0

Date are mean ± SD. ^$^ indicates a small difference between BA and PLA at 4 weeks. * indicates a small difference between BA and PLA at 10 weeks.

## 4. Discussion

We were unable to detect substantial physiological or performance benefits of 10 weeks of beta-alanine supplementation on non-laboratory controlled real-world competition outcomes in elite/sub-elite swimmers. *Post-hoc* analysis using IPS as a covariate to ascertain any influence of swimming ability also resulted in similar effects. In fact, our results indicate unclear effect of beta-alanine on competition performance.

Between the National Championships and the baseline training set, our swimmers had a rest period of 2 weeks, which is sufficient to result in a partial loss of training-induced adaptations [19]. Following beta-alanine supplementation for 4 weeks, there was a short-term improvement in training set performance suggesting some promise in the scenario of returning to training at the end of a post-competition rest block. However, this warrants further investigation as this ergogenic potential of beta-alanine was no longer evident at 10 weeks. The small (apparently counterintuitive) reduction of 0.8 mmol/L in peak blood lactate concentration after the high-intensity training sets is likely explained by possible differences in the effects of beta-alanine relative to that of other buffers such as sodium bicarbonate. Increased post-exercise blood lactate concentrations commonly seen with sodium bicarbonate ingestion may not necessarily accompany beta-alanine supplementation [6,13] as lactate transport could be facilitated by sodium bicarbonate co-transporters [20]. An alternate explanation is that lactate production in the blood is the sum of production and clearance, so it could be speculated that beta-alanine supplementation promotes aerobic/mitochondrial adaptations that result in less lactate/H^+^ production [21].

From the survey, 59% of the swimmers were able to guess correctly whether they were on beta-alanine or placebo. While there could be a placebo effect, the beta-alanine supplemented swimmers who correctly guessed their treatment did not perform better that those who believed they were on placebo. 

A limitation of our study is that we were unable to measure changes in muscle carnosine levels either directly (it would be unrealistic to undertake biopsy procedures on elite athletes during a phase of competition preparation) or non-invasively (we did not have access to MRI facilities) [22]. However, despite the evidence that trained populations exhibit higher “baseline” concentrations of muscle carnosine [23], the effectiveness of beta-alanine supplementation is not influenced by initial muscle carnosine content. Furthermore, the total dosage we used was similar to the amount (~380 g *vs.* ~415 g) supplemented to physically active males which increased muscle carnosine concentration by ~80% in the vastus lateralis [5]. Therefore, we feel confident that the swimmers who received the active treatment in our study are likely to have achieved a substantial increase in muscle carnosine concentration. 

Despite the likely increase in muscle buffering capacity associated with elevated carnosine content, there was no substantial effect on performance during competition in our study. This finding is in agreement with an investigation of elite 400-m runners where carnosine loading via beta-alanine supplementation did not improve indoor 400-m race time [6]. 

This study also examined the effect of beta-alanine supplementation on training sets requiring maximal efforts in a fatigued state within a controlled environment. There was a short-term superior improvement in training set times at 4 weeks but not 10 weeks. As the baseline test set was performed upon the return from two weeks of rest after the Australian Swimming Championships, beta-alanine supplementation could play a potential role in enhancing training outcomes after a post-competition rest block. However, the absence of an ergogenic effect after 10 weeks was in contrast to a similar study where subjects were able to increase total work done from a single bout of high-intensity cycling by 13% at 4 weeks and a further 3.2% at 10 weeks post beta-alanine supplementation [5]. It should be noted that we did not control for training load or intensity in our study and therefore, the absence of further improvements at 10 weeks of beta-alanine supplementation could also be washed out by a much larger effect of training adaptations. To accompany our findings, recent investigations within controlled settings examining 4 weeks of beta-alanine supplementation also reported a lack of ergogenic effects on a repeated team sport sprint protocol [24] and time to exhaustion in supra-maximal sprints [25].

A recent meta-analysis discussed divergent research findings from studies utilizing “capacity” and “performance” type protocols. Hobson and colleagues found that beta-alanine supplementation had a moderate effect on exercise capacity while having no benefit on measures of exercise performance [26] due to the employment of pacing strategies. To this end, our results seem to echo this rationale. In our high-intensity training “capacity” sets, designed to induce maximal cellular stress, we saw a small transient benefit of beta-alanine supplementation. Conversely we saw no benefit of chronic beta-alanine intake on our competition component, a measure of true “performance”. 

While the effect of beta-alanine supplementation on the performance of elite athletes within applied settings remains inconclusive, we note the parallels between this emerging literature on beta-alanine and that of other more established supplements. For example, studies involving creatine supplementation in elite swimmers have also reported a similar lack of enhancement of race performance in simulated or real competitions, despite clear evidence that creatine loading offers physiological and performance benefits to single or repeated bouts of exercise [27,28]. 

The absence of detectable effects in such studies most likely relates to the inherent variability in real-world swimming performance (in both training and competition) due to the influence of factors such as prior training, cumulative fatigue, diet and changes in technique [29]. Our study design did not attempt to control these variables since the logistics involved in standardizing training, diet and other confounding factors were deemed impractical in a multi-centre trial involving subjects located in 4 states and over 14 training squads. Moreover, we were interested to see if the effects of beta-alanine supplementation were sufficiently robust to be detectable even against this background noise. Indeed, the marketing of supplements usually claims that performance benefits are of such clear magnitude. It is also likely that the swimmers involved in our study completed training programs as dictated by their coaches based on their previous history or known ability to complete or respond to various sessions. Therefore, if the ergogenic benefit of beta-alanine is to allow the athlete to train harder, further research should be designed to allow modifications in training load to utilize the enhanced physiological mechanisms associated with muscle carnosine.

Other unforeseen factors such as injuries and sickness are also inevitable in real-world settings. For this instance, only 21 swimmers completed both the competition and training aspects of our study. While this scenario highlights the challenge in undertaking longitudinal research on dietary supplements, it is presumably indicative of the likely outcomes of supplement use in real life practice. Here, the effects of dietary supplements, even those which have evidence of small ergogenic benefit under controlled conditions, are less important or detectable in comparison to the moderate to large effects of fundamental factors such as well-chosen eating practices, consistent training, absence of illness and injury, and recovery strategies.

To our knowledge, this study is the first to investigate the ergogenic potential of beta-alanine supplementation in an elite athlete population within an applied non-laboratory controlled training and competitive setting. In conclusion, more research is required to clarify how the results of laboratory-based studies showing ergogenic benefits of beta-alanine can be transferred effectively into improved real-world training and competitive performance. 
